# Probabilistic multi-item inventory model with varying mixture shortage cost under restrictions

**DOI:** 10.1186/s40064-016-2962-2

**Published:** 2016-08-16

**Authors:** Hala A. Fergany

**Affiliations:** Department of Mathematics, Faculty of Science, Tanta University, Tanta, Egypt

**Keywords:** Probabilistic inventory model, Multi-item, Varying mixture shortage, Stochastic lead time demand

## Abstract

This paper proposed a new general probabilistic multi-item, single-source inventory model with varying mixture shortage cost under two restrictions. One of them is on the expected varying backorder cost and the other is on the expected varying lost sales cost. This model is formulated to analyze how the firm can deduce the optimal order quantity and the optimal reorder point for each item to reach the main goal of minimizing the expected total cost. The demand is a random variable and the lead time is a constant. The demand during the lead time is a random variable that follows any continuous distribution, for example; the normal distribution, the exponential distribution and the Chi square distribution. An application with real data is analyzed and the goal of minimization the expected total cost is achieved. Two special cases are deduced.

## Backround

The multi-item, single source inventory system is the most general procurement system which may be described as follows; an inventory of n-items is maintained to meet the average demand rates designated $\bar{D}_{1} ,\bar{D}_{2} ,\bar{D}_{3} , \ldots \ldots \bar{D}_{n}$. The objective is to decide when to procure each item, how much of each item to procure, in the light of system and cost parameters.

Hadley and Whiten (1963) treated the unconstrained probabilistic inventory models with constant unit of costs. Fabrycky and Banks (1965) studied the multi-item multi source concept and the probabilistic single-item, single source (SISS) inventory system with zero lead-time, using the classical optimization. Abou-El-Ata and Kotb (1996), Abou-El-Ata et al. 2003) studied multi-item EOQ inventory models-with varying costs under two restrictions. Moreover, Fergany and El-Saadani (2005, 2006; Fergany et al. 2014) treated constrained probabilistic inventory models with continuous distributions and varying costs.

The two basic questions that any continuous review $\left\langle {{\text{Q}},\;{\text{r}}} \right\rangle$ inventory control system has to answer are; when and how much to order. Over the years, hundreds of papers and books have been published presenting models for doing this under a wide variety of conditions and assumptions. Most authors have shown that the demand that cannot be filled from stock then backordered or the lost sales model are used. Several $\left\langle {{\text{Q}},\;{\text{r}}} \right\rangle$ inventory models with mixture of backorders and lost were proposed by Ouyang et al. (1996), Montgomery et al. (1973) and Park (1982). Also, Zipkin (2000) shows that demands occurring during a stockout period are lost sales rather than backorders.

**Fig. 1:**
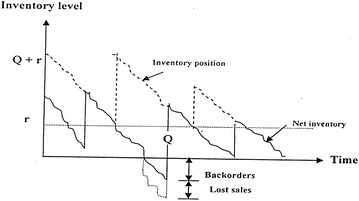
The inventory model

In this paper, we investigate a new probabilistic multi-item single-source (MISS) inventory model with varying mixture shortage cost (backorder and lost sales) as shown in Fig. 1 under two restrictions. One of them is on the expected varying backorder cost and the other one the expected varying lost sales cost. The optimal order quantity $Q_{i}^{*}$, the optimal reorder point $r_{i}^{*}$ and the minimum expected total cost [min E (TC)] are obtained. Moreover, two special cases are deduced and an application with real data is analyzed.

## The following notations are adopted for developing the model

- $\left\langle {Q,\,r} \right\rangle$ = the continuous review inventory system
- MISS = The Multi-item single-source,
- $D_{i}$ = The demand rate of the *i*th item per period,
- $\bar{D}_{i}$ = The expected demand rate of the *i*th item per period,
- $Q_{i}$ = The order quantity of the *i*th item per period,
- $Q_{i}^{*}$ = The optimal order quantity of the *i*th item per period,
- $r_{i}$ = The reorder point of the *i*th item per period,
- $r_{i}^{*}$ = The optimal reorder point of the *i*th item per period,
- $\bar{n}_{i}$ = The expected number order of the *i*th item per period,
- $L_{i}$ = The lead-time between the placement of an order and its receipt of the *i*th item,
- $\bar{L}_{i}$ = The average value of the lead time $L_{i}$,
- $x_{i}$ = The random variables represent the lead time demand of the *i*th item per period,
- $f\left( {x_{i} } \right)$ = The probability density function of the lead time demands,
- $E\left( {x_{i} } \right)$ = The expected value of $x_{i}$,
- $r_{i} - x_{i}$ = The random variable represents the net inventory when the procurement quantity arrives if the lead-time demand x ≤ r,
- $\bar{H}_{i}$ = The average on hand inventory of the *i*th item per period
- $R\left( r \right) = p\left( {x_{i} > r} \right)$ = The probability of shortage = the reliability function,
- $\bar{S}\left( {r_{i} } \right)$ = The expected shortage quantity per period
- $c_{oi}$ = The order cost per unit of the *i*th item per period,
- $c_{hi}$ = The holding cost per unit of the *i*th item per period,
- $c_{si}$ = The shortage cost per unit of the *i*th item per period,
- $c_{bi}$ = The backorder cost per unit of the *i*th item per period,
- $c_{li}$ = The lost sales cost per unit of the *i*th item per period,
- $c_{si} (n)$ = The varying shortage cost of the *i*th item per period,
- $\Phi_{D} \left( t \right)$ = The characteristic function of demand,
- $\Phi_{x} \left( t \right)$ = The characteristic function of lead time demand x,
- $\beta$ = A constant real number selected to provide the best fit of estimated expected
- cost function,
- $\gamma_{i}$ = The backorder fraction of the *i*th item, $0 < \gamma_{i} < 1$,
- *E (OC)* = The expected order (procurement) cost per period,
- *E (HC)* = The expected holding (carrying) cost per period,
- *E (SC*) = The expected shortage cost per period,
- *E (BC)* = The expected backorder cost per period,
- *E (LC)* = The expected lost sales cost per period,
- *E (TC)* = The expected total cost function,
- Min *E (TC)* = The minimum expected total cost function.
- $K_{bi}$ = The limitation on the expected annual varying backorder cost for
- backorder model of the *i*th item,
- $K_{li}$ = The limitation on the expected annual varying lost sales cost for
- lost sales model of the *i*th item.

## Mathematical model

We will study the proposed model with varying mixture shortage cost constraint when the demand D is a continuous random variable, the lead-time L is constant and the distribution of the lead time demand (demand during the lead time) is known.

It is possible to develop the expected annual total cost as follows:

$${\text{E}}\left( {{\text{Total}}\;{\text{Cost}}} \right) = \sum\limits_{i = 1}^{m} {[E(Order\,\text{Cos} t)\, + \,E(Holding\,\text{Cos} t)\, + E(Shortage\,\text{Cos} t)} ]$$

i.e.

$$E\left[ {TC\left( {Q,r} \right)} \right] = \sum\limits_{i = 1}^{m} {\left[ \begin{aligned} &\left( {c_{oi} \left( {\frac{{\bar{D}_{i} }}{{Q_{i} }}} \right)} \right) + c_{hi} \left( {\frac{{Q_{i} }}{2} + r_{i} - E\left( {x_{i} } \right)} \right) + c_{bi} \gamma \left( {\frac{{\bar{D}_{i} }}{{Q_{i} }}} \right)^{{^{\beta + 1} }} \int\limits_{r}^{\infty } {\left( {x_{i} - r_{i} } \right)f\left( {x_{i} } \right)dx_{i} } \hfill \\& + \left( {c_{li} \left( {\frac{{\bar{D}_{i} }}{{Q_{i} }}} \right)^{\beta + 1} + c_{hi} } \right)\left( {1 - \gamma_{i} } \right)\int\limits_{r}^{\infty } {\left( {x_{i} - r_{i} } \right)f\left( {x_{i} } \right)dx_{i} } \hfill \\ \end{aligned} \right]}$$

where; $$\int_{r}^{\infty } {\left( {x_{i} - r_{i} } \right)f\left( {x_{i} } \right)dx_{i} = \bar{S}(r_{i} )}$$

The objective is to minimize the expected annual total cost E [TC (Q, r)] under two constraints:

$$c_{bi} \gamma_{i} \,\left( {\frac{{\bar{D}_{i} }}{{Q_{i} }}} \right)^{\beta + 1} \bar{S}\left( {r_{i} } \right) - K_{bi} \le 0$$

$$c_{li} \,\left( {1 - \gamma_{i} } \right)\,\left( {\frac{{\bar{D}_{i} }}{{Q_{i} }}} \right)^{\beta + 1} \bar{S}\left( {r_{i} } \right) - K_{li} \le 0$$

To solve this primal function which is a convex programming problem, let us write the previews equations in the following form:

1 $$E\left[ {TC\left( {Q,r} \right)} \right] = \sum\limits_{i = 1}^{m} {\left[ \begin{aligned} &\left( {c_{oi} \frac{{\bar{D}_{i} }}{{Q_{i} }}} \right) + c_{hi} \left( {\frac{{Q_{i} }}{2} + r_{i} - E\left( {x{}_{i}} \right)} \right) \hfill \\& + \left( {c_{bi} \gamma \left( {\frac{{\bar{D}_{i} }}{{Q_{i} }}} \right)^{\beta + 1} \bar{S}\left( {r_{i} } \right)} \right) + \left( {c_{li} \left( {\frac{{\bar{D}_{i} }}{{Q_{i} }}} \right)^{\beta + 1} + c_{hi} } \right)\left( {1 - \gamma_{i} } \right)\bar{S}\left( {r_{i} } \right) \hfill \\ \end{aligned} \right]}$$

Subject to:

2 $$\left. \begin{aligned} c_{bi\,} \gamma_{i} \,\left( {\frac{{\bar{D}_{i} }}{{Q_{i} }}} \right)^{\beta + 1\,} \bar{S}(r_{i} ) - K_{bi} \, \le 0 \hfill \\ c_{li\,} \left( {1 - \gamma_{i} } \right)\,\left( {\frac{{\bar{D}_{i} }}{{Q_{i} }}} \right)^{\beta + 1\,} \bar{S}(r_{i} ) - K_{li} \, \le 0 \hfill \\ \end{aligned} \right\}$$

To find the optimal values $Q^{*} \,{\text{and}}\,r^{*}$ which minimize Eq. (1) under the constraints (2), the Lagrange multiplier technique is used as follows:

3 $$\begin{aligned} L\left( {Q_{i} ,r_{i} ,\lambda_{i1} \lambda_{i2} } \right) & = \sum\limits_{i = 1}^{m} {\left[ {\frac{{\bar{D}_{i} }}{{Q_{i} }} + c_{hi} \left\{ {\frac{{Q_{i} }}{2} + r_{i} - E\left( {x_{i} } \right)} \right\} + c_{bi} \gamma_{i} \left( {\frac{{\bar{D}_{i} }}{{Q_{i} }}} \right)^{\beta + 1} \bar{S}\left( {r_{i} } \right)} \right.} \\ & \quad + \left\{ {c_{li} \left( {\frac{{\bar{D}_{i} }}{{Q_{i} }}} \right)^{\beta + 1} + c_{hi} } \right\}\left( {1 - \gamma_{i} } \right)\bar{S}\left( {r_{i} } \right) + \lambda_{1i} \left\{ {c_{bi} \gamma_{i} \left( {\left( {\frac{{\bar{D}_{i} }}{{Q_{i} }}} \right)^{\beta + 1} } \right)\bar{S}\left( {r_{i} } \right) - k_{bi} } \right\} \\ & \quad \left. { + \lambda_{2i} \left\{ {C_{li} \left( {1 - \gamma_{i} } \right)\left( {\frac{{\bar{D}_{i} }}{{Q_{i} }}} \right)^{\beta + 1} \bar{S}\left( {r_{i} } \right) - k_{li} } \right\}} \right] ,\end{aligned}$$

where $\lambda_{1i} ,\,\lambda_{2i}$ are the Lagrange multipliers.

The optimal values $Q_{i} \;{\text{and}}\;r_{i}$ can be calculated by setting each of the corresponding first partial derivatives of Eq. (3) equal to zero.

i.e.

$$\frac{\partial L}{{\partial Q_{i} }} = 0\quad \frac{\partial L}{{\partial r_{i} }} = 0,$$

then we obtain:

4 $$C_{bi} Q_{i}^{*\beta + 2} - 2C_{oi} Q_{i}^{*\beta } - 2A\left( {\beta + 1} \right)\bar{S}\left( {r_{i} } \right) = 0,$$

5 $$R\left( {r_{i}^{*} } \right) = \left[ {\frac{{C_{hi} Q_{i}^{*B + 1} }}{{A + C_{hi} \left( {1 - \gamma_{i} } \right)Q_{i}^{*\beta + 1} }}} \right]$$

where $$A = \bar{D}_{i}^{\beta + 1} \left[ {\gamma_{i} \,C_{hi} \left( {1 + \lambda_{1i} } \right) + \left( {1 - \gamma_{i} } \right)C_{li} \left( {1 + \lambda_{2i} } \right)} \right]$$

Clearly, there is no closed form solution of Eqs. (4), (5).

### Mathematical derivation of the lead time demand

 The lead time demand $X$ is the total demand D which accrue during the lead time L. Consider that the lead time is a constant number of periods and demand is random variable.

Then,

$$X = \sum\limits_{i = 1}^{L} {D_{i} } ,\quad i = 1,2, \ldots \ldots ,L$$

To determine the distribution of the lead time demand X: consider the characteristic function of $X$ and D are related as:

$$\Phi_{x} \left( t \right) = \prod\limits_{i = 1}^{L} {\Phi_{D} \left( t \right)} = [\Phi_{D} \left( t \right)]^{L}$$

We can deduce the corresponding distribution of the lead time demand X when the demand follows many continuous distributions. Consider X follows the normal distribution, the exponential distribution and the Chi square distribution.

### The demand follows the normal distribution

If the demand D have the normal distribution with parameters $\mu ,\sigma$,

$$f\left( D \right) = \frac{1}{{\sigma \sqrt {2\pi } }}e^{{ - \frac{1}{2}\left[ {\frac{D - \mu }{\sigma }} \right]^{2} }} ,\quad - \infty < D < \infty ,\, - \infty < \mu < \infty ,\,\sigma > 0$$

Then the lead time demand follows the normal distribution with parameters $\mu L,\;L\sigma^{2}$

$$f\left( x \right) = \frac{1}{{\sigma \sqrt {2\pi L} }}e^{{ - \frac{1}{2L}\left[ {\frac{x - \mu L}{\sigma }} \right]^{2} }} ,\quad - \infty < x < \infty ,\, - \infty < \mu \,L < \infty ,\,\sigma \,L > 0$$

Also: $$R(r){\mkern 1mu} = \int_{r}^{\infty } {f(x)d(x)}$$ i.e.

$$R\left( r \right) = 1 - \phi \left( {\frac{{r - \mu L}}{{\sigma \sqrt L }}} \right) = \varphi \left[ {\frac{{r - \mu L}}{{\sigma \sqrt L }}} \right]$$

and

6 $$\bar{S}\left( r \right) = \sigma \sqrt L \,\Psi \left( {\frac{r - \mu L}{\sigma \sqrt L }} \right) + \left( {\mu L - r} \right)\,\varphi \left( {\frac{r - \mu L}{\sigma \sqrt L }} \right)$$

where

$$\Psi \left( {\frac{r - \mu L}{\sigma \sqrt L }} \right) = \frac{1}{{\sqrt {2\pi } }}\int\limits_{{\frac{r - \mu L}{\sigma \sqrt L }}}^{\infty } {y\,e^{{ - \frac{1}{2}y^{2} }} dy}$$

Hence, the expected annual total cost can be minimized mathematically by substituting from Eq. (6) into (4), (5) we get (7), (8)

7 $$C_{hi} Q_{i}^{*\beta + 2} - 2C_{oi} Q_{r}^{*\beta } - 2A\left( {\beta + 1} \right)\left[ {\sigma \sqrt L \Psi \left( {\frac{r - \mu L}{\sigma \sqrt L }} \right) + \left( {\mu L - r} \right)\varphi \left( {\frac{r - \mu L}{\sigma \sqrt L }} \right)} \right],$$

and

8 $$\phi \left( {\frac{r - \mu L}{\sigma \sqrt L }} \right) = \left[ {\frac{{C_{hi} Q_{i}^{*\beta + 1} }}{{C_{hi} \left( {1 - \gamma } \right)Q_{i}^{*\beta + 1} + A}}} \right]$$

### The demand follows the exponential distribution

If the demand D have the exponential distribution with parameter $\alpha$,

$$f\left( x \right) = \alpha \,e^{ - \alpha \,D} ,\quad 0\, < \,D\, < \,\infty \,,\,\alpha \, > \,0$$

Then, lead time demand follows the Gamma distribution with parameters $L,\alpha$

$$f\left( x \right)\, = \frac{{\alpha^{L} }}{\Gamma \left( L \right)}\;x^{L - 1} \;e^{ - \alpha \,x} ,\quad 0 < \,x\, < \,\infty ,\,L\,> \,0,\,\alpha \, > \,0,$$

also $$R\left( r \right) = \frac{{\alpha^{L} }}{\Gamma \left( L \right)}\int\nolimits_{r}^{\infty } {x^{L - 1} } e^{ - \alpha x} dx$$ then, $$R\left( r \right) = \sum\limits_{i = 0}^{L - 1} {\left[ {\frac{{\left( {\alpha r} \right)^{i} e^{ - \alpha r} }}{i\,!\,}} \right]}$$,

$$\bar{S}\left( r \right) = \int\limits_{r}^{\infty } {\left( {x - r} \right)} \,f\left( x \right)dx = \frac{{\alpha^{L} }}{\Gamma \left( L \right)}\int\limits_{r}^{\infty } {\left( {x - r} \right)x^{L - 1} } e^{ - \alpha r} dx = \frac{{\alpha^{L} }}{\Gamma \left( L \right)}\int\limits_{r}^{\infty } {x^{L} } e^{ - \alpha r} dx - rR\left( r \right)$$

9 $$\bar{S}\left( r \right) = \frac{L}{\alpha }\,\,\left[ {\sum\limits_{i = 0}^{L} {\left\{ {\frac{{\left( {\alpha r} \right)^{i} e^{ - \alpha r} }}{i\,!\,}} \right\}} } \right] \,- \,r\,\,\left[ {\sum\limits_{i = 0}^{L - 1} {\left\{ {\frac{{\left( {\alpha r} \right)^{i} e^{ - \alpha r} }}{i\,!\,}} \right\}} } \right]$$

Hence, the expected annual total cost can be minimized mathematically by substituting from Eq. (9) into (4), (5) we get (10), (11)

10 $$C_{hi} \,Q_{i}^{*\beta + 2} - 2\,C_{oi} \,Q_{i}^{*\beta } - 2\,A\,\,\left( {\beta + 1} \right)\,\left[ {\frac{L}{\alpha }\,\sum\limits_{i = 0}^{L} {\left\{ {\frac{{\left( {\alpha r} \right)^{i} e^{ - \alpha r} }}{i\,!\,}} \right\}} - r\sum\limits_{i = 0}^{L - 1} {\left\{ {\frac{{\left( {\alpha r} \right)^{i} e^{ - \alpha r} }}{i\,!\,}} \right\}} } \right],$$

and

11 $$\varphi \left( {\frac{r - \mu L}{\sigma \sqrt L }} \right) = \left[ {\frac{{C_{hi} \,Q_{i}^{*\beta + 1} }}{{C_{hi} \,\left( {1 - \gamma } \right)\,Q_{i}^{*\beta + 1} + A}}} \right] = \sum\limits_{i = 0}^{L - 1} {\left[ {\frac{{\left( {\alpha \,r} \right)^{i} e^{ - \alpha r} }}{i\,!}} \right]}$$

### The demand follows the Chi square distribution

If the demand D follows Chi-squire distribution with parameter $\frac{\eta }{2}$

$$f\left( D \right) = \frac{1\,}{{2^{{\frac{\eta }{2}}} \Gamma \left( {\frac{\eta }{2}} \right)}}D^{{\frac{\eta }{2} - 1}} ,\quad 0 < D < \infty ,\,\frac{\eta }{2} > 0$$

Then lead time demand X follows the Chi-squire distribution with parameters $\frac{L\eta }{2}$

$$f\left( x \right) = \frac{1}{{2^{{\frac{L\eta }{2}}} \Gamma \left( {\frac{L\eta }{2}} \right)}}\,x^{{\frac{L\,\eta }{2} - 1}} \quad 0 < x < \infty ,\;\frac{L\eta }{2} > 0,$$

also

$$R\left( r \right) = \sum\limits_{i = 0}^{{\frac{L\eta }{2} - 1}} {\left[ {\frac{{\left( {\frac{r}{2}} \right)^{i} \,e^{{ - \frac{r}{2}}} }}{i\,!\,}} \right]} ,$$

and

12 $$\bar{S}\left( r \right) = L\eta \,\left[ {\sum\limits_{i = 0}^{{\frac{L\eta }{2}}} {\left\{ {\frac{{\left( {\frac{r}{2}} \right)^{i} e^{{ - \frac{r}{2}}} }}{i\,!\,}} \right\}} } \right] - r\left[ {\sum\limits_{i = 0}^{{\frac{L\eta }{2} - 1}} {\left\{ {\frac{{\left( {\frac{r}{2}} \right)^{i} e^{{ - \frac{r}{2}}} }}{i\,!\,}} \right\}} } \right]$$

Hence, the expected annual total cost can be minimized mathematically by substituting from Eq. (12) into (4), (5) we get (13), (14):

13 $$C_{hi} \,Q_{i}^{*\beta + 2} - 2\,C_{oi} Q_{i}^{*\beta } - 2\,A\,\left( {\beta + 1} \right)\,\left[ {L\eta \,\sum\limits_{i = 0}^{{\frac{L\eta }{2}}} {\left\{ {\frac{{\left( {\frac{r}{2}} \right)^{i} e^{{ - \frac{r}{2}}} }}{i\,!}} \right\}} - r\,\sum\limits_{i = 0}^{{\frac{L\eta }{2}}} {\left\{ {\frac{{\left( {\frac{r}{2}} \right)^{i} e^{{ - \frac{r}{2}}} }}{i\,!\,}} \right\}} } \right],$$

and

14 $$\varphi \left( {\frac{r - \mu L}{\sigma \sqrt L }} \right) = \left[ {\frac{{C_{hi} \,Q_{i}^{*\beta + 1} }}{{C_{hi} \left( {1 - \gamma } \right)Q_{i}^{*\beta + 1} + A}}} \right] = \sum\limits_{i = 0}^{{\frac{L\eta }{2} - 1}} {\left[ {\frac{{\left( {\frac{r}{2}} \right)^{i} e^{{ - \frac{r}{2}}} }}{i\,!}} \right]}$$

## Special cases

Two special cases of the proposed model are deduced as follows;

### **Case 1**

$Let\;\gamma_{i} = 0,\;\beta = 0\;and\;K_{bi} \to \infty \; \Rightarrow c_{s} \left( {\bar{n}} \right)^{\beta } = c_{s} \;and\;\lambda_{i} = 0.$ Thus Eqs. (4) and (5) become:

$$Q^{*} = \sqrt {\frac{{2\bar{D}\left( {c_{o} + c_{l} \bar{S}\left( r \right)} \right)}}{{c_{h} }}} \; {\text{and}}\;R\left( {r^{*} } \right) = \frac{{c_{h} Q^{*} }}{{c_{h} Q^{*} + c_{l} \bar{D}}}$$

This is the unconstrained lost sales continuous review inventory model with constant units of cost, which are the same results as in Hadley and Whiten (1963).

### **Case 2**

$$Let\,\gamma_{i} = 1\,\beta = 0\,and\,K_{li} \to \infty \, \Rightarrow c_{s} \left( {\bar{n}} \right)^{\beta } = c_{s} \,and\,\,\lambda_{i} = 0\,.\,$$

Thus Eqs. (4) and (5) become:

$$Q^{*} = \sqrt {\frac{{2\bar{D}\left( {c_{o} + c_{b} \bar{S}\left( r \right)} \right)}}{{c_{h} }}} \;{\text{and}}\;R\left( {r^{*} } \right) = \frac{{c_{h} }}{{c_{b} \bar{D}}}Q,$$

This is the unconstrained backorders continuous review inventory model with constant unit costs, which coincide with the result of Hadley and Whiten (1963).

## Applications

A company for ready clothes produces three Items [Trousers: I, Shirt: II, and Jacket: III] of seasonal products (production takes two cycles and each cycle lasts for 6 months). Table 5 in Appendix shows the order quantity and the demand rate during the interval 2004–2008. But for some un expected reasons in some cycles, the company faces shortage and it has to pay penalty at least 1 % for month for backorder and 3 % for lost sale. Table 1 shows the maximum cost allowed for backorder $K_{b}$, lost sales $K_{L}$ and their fractions. Hence, the company wishes to put an optimal policy for production to minimize the expected total cost.

**Table 1:** The Maximum cost allowed (the limitations) for both backorder, lost sales and their fractions

Items	Costs			
	K_b_	K_L_	*γ*	$\left( {1 - \gamma } \right)$
Item (I)	1680	13,720	0.56	0.44
Item (II)	1800	9300	0.70	0.30
Item (III)	1052	10,820	0.67	0.33

### Solution

By using SPSS program, One-Sample Kolmogorov–Smirnov Test, the demand for the three Items is fitted to normal distribution, where Table 2 shows the K-S statistic with their *P* values. Table 3 shows the average units cost for each item 2004–2008

**Table 2:** One-sample Kolmogorov–Smirnov test of the demands

	D1	D2	D3
N	48	48	48
Normal parameters^a^			
Mean	1.07E4	1.12E4	6109.38
SD	2.300E3	2.258E3	3.603E3
Most extreme differences			
Absolute	0.193	0.180	0.196
Positive	0.091	0.109	0.176
Negative	−0.193	−0.180	−0.196
Kolmogorov–Smirnov Z	1.335	1.245	1.359
Asymp. Sig. (2-tailed)	0.057	0.090	0.050

^a^Test distribution is normal

**Table 3:** The average units cost for each item 2004–2008

Items	Costs			
	$c_{o}$	$c_{h}$	Shortage cost	
			$c_{b}$	$c_{l}$
Item (I)	2.23	7.898	0.90	9.350
Item (II)	2.14	7.567	1.10	13.254
Item (III)	9.77	34.542	3.28	68.460

The optimal values $Q^{*}$ and $r^{*}$ for three items can be found by using (7) and (8) respectively. The iterative procedure will be used to solve the equations.

Use the following numerical procedure:

* Step 1: Assume that $\bar{S}\, = \,0$ and $r = E(x)$, then from Eq. (7) we have: $Q_{0} = \sqrt {\frac{{2c_{oi} \bar{D}_{i} }}{{c_{hi} }}}$
* Step 2: Substituting $Q_{o}$ into Eq. (8) we obtain $r_{0}$
* Step 3: Substituting by $r_{0}$ from step 2 into Eq. (7) we can deduce $Q_{1}$
* Step 4: the procedure is to change the values of $\lambda_{i}$ in step 2 and step 3 until the smallest value of $\lambda_{i} > 0$ is found such that the constraint varying shortage for the different values of *β*.

**Fig. 2:**
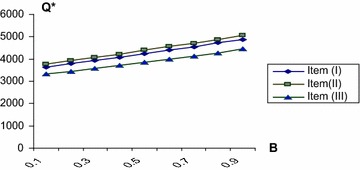
The optimal values of Q* against *β*

**Fig. 3:**
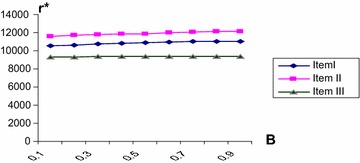
The optimal values of r* against *β*

**Fig. 4:**
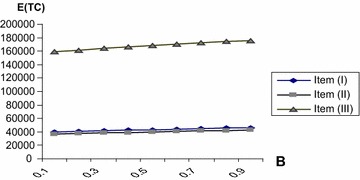
The optimal values of E(TC) against *β*

The numerical computation are done by using mathematica program for three items at different values of *β*, Table 4 shows the optimal values $Q^{*},r^{*}$ E(TC) and min E(TC) at different values of $\beta$. Hence we can draw the optimal routes of $Q^{*},\,r^{*}$ and E (TC) against *β* for all three items as shown in Figs. 2, 3 and 4. It is evident that the min E(TC) is achieved at minimum value for *β*.

**Table 4:** The optimal values of $Q^{*} ,r^{*}$ and min E (TC) at different values of *β*

*β*	Item 1					Item 2					Item 3				
	$\lambda_{1}^{*}$	$\lambda_{2}^{*}$	$Q^{*}$	$r^{*}$	min E (TC1)	$\lambda_{1}^{*}$	$\lambda_{2}^{*}$	$Q^{*}$	$r^{*}$	min E (TC2)	$\lambda_{1}^{*}$	$\lambda_{2}^{*}$	$Q^{*}$	$r^{*}$	min E (TC3)
0.1	0.02	0.021	3635.43	10,543	39,538	0.001	0.012	3758	1161	36,431	0.14	0.012	3322	9282	159,060
0.2	0.024	0.025	3786.93	10,635	40,586	0.001	0.021	3926	11,699	37,443	0.13	0.18	3430	9323	161,426
0.3	0.025	0.027	3931.32	10,727	41,582	0.002	0.022	4071	11,789	38,400	0.13	0.19	3584	9364	164,554
0.4	0.032	0.034	4083.49	10,819	42,467	0.002	0.027	4210	11,879	39,256	0.13	0.19	3717	9384	166,737
0.5	0.039	0.040	4246	10,888	43,302	0.004	0.042	4404	11,857	39,603	0.12	0.19	3852	9384	168,639
0.6	0.042	0.043	4413.04	10,934	44,124	0.005	0.052	4554	11,992	40,902	0.12	0.19	3990	9384	170,104
0.7	0.043	0.044	4554.17	11,003	44,886	0.008	0.063	4719	12,069	41,634	0.12	0.19	4124	9405	172,461
0.8	0.048	0.046	4730.91	11,026	45,598	0.01	0.068	4881	12,104	42,323	0.12	0.19	4261	9405	174,186
0.9	0.049	0.048	4876.67	11,026	45,865	0.01	0.071	5056	12,149	43,008	0.13	0.19	4455	9364	175,779

## Conclusion

Upon studying the probabilistic multi item invetory model with varying mixture shortage cost under two restrictions using the Lagrange mulipliers technique, the optimal order quntity $Q^{*}$ and the optimal reorder point $r^{*}$ are introduced. Then, the minimum expected total cost min E(TC) for multi items are deduced. Three curves $Q^{*}$, $r^{*}$ and min E(TC) are displayed to illustate them for multi items against the different values of *β*. Finally, the min E(TC) is achieved at minimum value for *β*.

## Appendix

See Table 5.

**Table 5:** The actual inventory quantity and demand rate, from May 2004 to April 2008

Year	No. of cycle	Month	Item 1		Item 2		Item 3	
			Q1	D1	Q2	D2	Q3	D3
2004	1	May	5800	6000	10,500	10,500	8000	900
		June	9000	8000	9000	10,000	5500	500
		July	11,800	12,000	12,000	12,000	8000	900
		Aug	11,800	12,000	12,000	12,500	6000	500
		Sept.	8000	8500	10,000	9000	4000	400
		Oct.	7200	7000	7500	7000	3000	400
	2	Nov.	10,000	10,000	10,000	10,500	5500	500
		Dec.	11,000	12,000	9000	9000	5500	500
2005		Jan.	12,800	12,800	11,000	11,000	5000	550
		Feb.	11,000	10,000	7500	7500	4000	500
		March	6000	6500	12,500	12,500	5000	500
		April	9500	8500	13,000	12,500	7000	600
	3	May	12,000	12,000	11,000	12,000	9500	10,000
		June	12,000	12,500	10,000	9000	6500	6000
		July	8500	9000	12,500	12,800	9000	10,000
		Aug.	7000	7500	17,000	16,000	7000	6000
		Sept.	11,000	12,000	9000	10,000	5000	5000
		Oct.	13,400	11,000	7800	8000	4000	5000
	4	Nov.	12,850	13,500	12,500	12,000	6500	6000
		Dec.	12,830	13,000	11,000	12,000	6500	
2006		Jan.	12,850	12,500	11,850	10,500	7000	7500
		Feb.	12,830	11,850	6830	8000	6000	7000
		March	12,820	12,000	11,820	12,500	7000	7000
		April	10,730	11,030	12,730	12,230	9000	8000
	5	May	6500	7000	11,500	12,000	10,000	11,000
		June	9800	8500	10,000	9500	7500	7000
		July	12,500	13,000	12,800	12,950	10,000	11,000
		Aug.	12,200	13,000	17,000	16,000	8500	7000
		Sept.	9000	8600	9000	9500	6000	6000
		Oct.	7000	7300	8500	8750	5000	6000
	6	Nov.	10,000	12,000	13,000	12,000	7500	7000
		Dec.	12,000	10,500	11,500	12,500	7500	7000
		Jan.	13,000	14,000	12,000	11,000	8000	8500
		Feb.	13,000	13,000	7000	8000	7000	8000
		March	13,000	12,000	12,000	13,000	8000	8000
		April	11,000	10,000	13,000	13,000	10,000	9000
		May	7000	7000	12,000	13,000	11,500	12,000
		June	10,000	11,000	10,000	9000	8500	8000
		July	13,000	13,000	13,000	14,000	11,000	12,000
		Aug.	12,000	13,000	17,000	16,000	9000	8000
		Sept.	9000	9000	11,000	9000	7000	7000
		Oct.	10,000	8000	8000	9000	7000	7000
	8	Nov.	10,000	12,000	13,000	12,000	8500	8000
		Dec.	12,000	10,000	11,500	12,000	8500	8000
2008		Jan.	14,000	14,500	12,500	12,000	9000	9500
		Feb.	13,000	13,200	8000	7500	8000	9000
		March	13,000	13,000	13,000	13,000	9000	9000
		April	11,000	10,000	14,000	14,000	11,000	10,000
